# Correction: Updated Integrated Analysis of the Efficacy and Safety of Entrectinib in Patients with *NTRK* Fusion–Positive Solid Tumors

**DOI:** 10.1158/1078-0432.CCR-22-1108

**Published:** 2022-05-13

**Authors:** George D. Demetri, Filippo De Braud, Alexander Drilon, Salvatore Siena, Manish R. Patel, Byoung Chul Cho, Stephen V. Liu, Myung-Ju Ahn, Chao-Hua Chiu, Jessica J. Lin, Koichi Goto, Jeeyun Lee, Lyudmila Bazhenova, Thomas John, Marwan Fakih, Sant P. Chawla, Rafal Dziadziuszko, Takashi Seto, Sebastian Heinzmann, Bethany Pitcher, David Chen, Timothy R. Wilson, Christian Rolfo

In the earlier version of this article as it was published online on February 10, 2022 (1) and in the printed article, the disclosures of Salvatore Siena and Jeeyun Lee were missing. The HTML and PDF versions of this article were corrected in the online release of the April 1, 2022 issue (2). The publisher regrets the error.
